# Risk factors for early adverse outcomes after bovine jugular vein conduit implantation: influence of oversized conduit on the outcomes

**DOI:** 10.1093/icvts/ivac197

**Published:** 2022-07-27

**Authors:** Dong-Hee Kim, Young Kern Kwon, Eun Seok Choi, Bo Sang Kwon, Chun Soo Park, Tae-Jin Yun

**Affiliations:** Division of Pediatric Cardiac Surgery, Department of Thoracic and Cardiovascular Surgery, Asan Medical Center, University of Ulsan College of Medicine, Seoul, Korea; Division of Pediatric Cardiac Surgery, Department of Thoracic and Cardiovascular Surgery, Asan Medical Center, University of Ulsan College of Medicine, Seoul, Korea; Division of Pediatric Cardiac Surgery, Department of Thoracic and Cardiovascular Surgery, Asan Medical Center, University of Ulsan College of Medicine, Seoul, Korea; Division of Pediatric Cardiac Surgery, Department of Thoracic and Cardiovascular Surgery, Asan Medical Center, University of Ulsan College of Medicine, Seoul, Korea; Division of Pediatric Cardiac Surgery, Department of Thoracic and Cardiovascular Surgery, Asan Medical Center, University of Ulsan College of Medicine, Seoul, Korea; Division of Pediatric Cardiac Surgery, Department of Thoracic and Cardiovascular Surgery, Asan Medical Center, University of Ulsan College of Medicine, Seoul, Korea

**Keywords:** Bovine jugular vein conduit, Contegra, Right ventricular outflow tract, Explantation, Endocarditis

## Abstract

**OBJECTIVES:**

We investigated potential risk factors for early failure of bovine jugular vein conduit (Contegra^®^) implantation for right ventricular outflow tract (RVOT) reconstruction.

**METHODS:**

A single-centre retrospective review of 115 consecutive patients (54 males) who underwent RVOT reconstruction with Contegra between 2016 and 2019 was performed. Overall survival, explantation-free survival and freedom from significant RVOT lesions (valve regurgitation ≥ moderate or flow velocity ≥3.5 m/s) were investigated.

**RESULTS:**

Median age, body weight and Contegra diameter were 10.3 months [interquartile range (IQR) 5.7–26.9 months], 7.8 kg (IQR 6.3–12.4 kg) and 14 mm (IQR 12–16 mm), respectively. During the median follow-up duration of 25.1 months, there were 7 deaths, 34 significant RVOT lesions, 10 endocarditis episodes and 15 explantations. Overall survival and explantation-free survival at 3 years were 94.8% and 78.4%, respectively. Significant RVOT lesions (*n* = 34) comprised 20 stenoses, 8 regurgitations and 6 combined lesions. Freedom from significant RVOT lesions at 3 years was 62.6%. Cox regression identified higher indexed Contegra size (Contegra diameter/body weight, mm/kg) as the only risk factor for decreased time to explantation or death (hazard ratio 2.32, *P *<* *0.001) and time to significant RVOT lesions development (hazard ratio 2.75, *P *<* *0.001). The cut-off value of indexed Contegra size for significant RVOT lesions at 12 months was 1.905 mm/kg (sensitivity, 0.75; specificity, 0.78; area under the curve, 0.82).

**CONCLUSIONS:**

Outcomes of RVOT reconstruction using Contegra were acceptable. However, oversized Contegra should be avoided when possible.

**IRB APPROVAL DATE:**

26 October 2020.

**IRB REGISTRATION NUMBER:**

S2020-2505-0001

**Figure:**
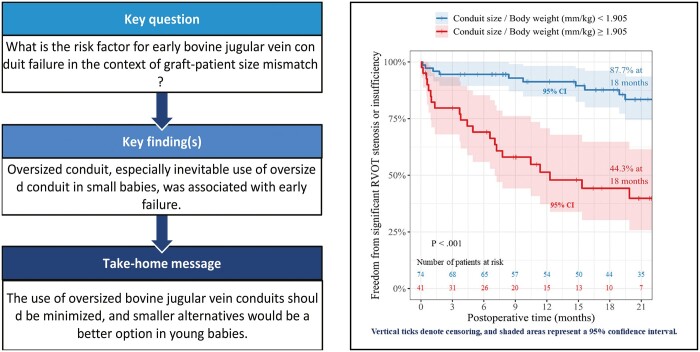

## INTRODUCTION

The surgical treatment of various congenital heart diseases frequently entails reconstruction of the right ventricular outflow tract (RVOT) using a conduit. Homograft has been widely used for RVOT reconstruction; however, limited homograft availability has led to the development of several alternatives [1, 2]. Surgically implantable bovine jugular vein conduit, commercially registered as Contegra (Medtronic Inc, Minneapolis, MN, USA), was introduced in 1999 as an alternative to homograft or other prosthetic conduits [3]. The use of Contegra has been increasingly popular ever since due to several advantages: it is readily available in various sizes (from 12 to 22 mm), easy to handle and it provides sufficient pre-valvular and post-valvular conduit length, which eliminates the need for additional patch augmentation in anastomotic sites [4, 5]. However, controversy over Contegra use has remained unresolved because of inconsistent clinical outcomes in the ensuing studies [6]. A number of Contegra advocates have reported promising outcomes [3, 7, 8], while others have asserted concerns about a high incidence of adverse outcomes, including distal anastomotic site stenosis [9], thrombus formation, aneurysmal changes [10], valvar insufficiency [6] and endocarditis [4–6, 9, 11, 12]. Smaller Contegra conduits [6, 13], younger recipient age at implantation and specific patient characteristics, such as the association of arbourization anomalies [14] or pulmonary arterial hypertension, have been proposed as risk factors for adverse outcomes [15]. Although smaller conduits tend to be used for smaller or younger patients, conduits may be oversized, adequately sized or undersized, depending on the ratio of conduit diameter to the patient’s body size. The advantages and disadvantages of oversized or undersized conduits are still under debate. We investigated the risk of adverse outcomes after Contegra implantation in the context of graft-patient size mismatch.

## PATIENTS AND METHODS

### Ethical statement

Data collection, collation and analysis were approved by the institutional review board (IRB No.: S2020-2505-0001) as of 26 October 2020, and the need for informed consent was waived because of the retrospective nature of the study.

### Patients

A single-centre retrospective study was performed for 115 consecutive patients who underwent RVOT reconstruction using Contegra between 2016 and 2019. All patients receiving their first Contegra implantation, including new RVOT reconstructions or replacements of other types of conduits, were included. Outcomes of interest were overall survival, Contegra explantation-free survival and the development of significant lesions in the reconstructed RVOT. Significant RVOT lesions comprised regurgitation (≥ moderate) and stenosis (flow velocity in the RVOT ≥ 3.5 m/s), and stenotic lesions were classified according to the level of stenoses (i.e. proximal anastomotic site, conduit *per se* and distal anastomotic site). Conduit explantation was performed for patients who developed haemodynamically significant stenosis that could not be alleviated by catheter intervention, significant regurgitation or infective endocarditis not yielding to medical treatment. Contegra diameter indexed to the patient’s body weight at the time of surgery (i.e., indexed Contegra size) was used as a surrogate for conduit-patient size mismatch. Contegra diameter was also translated into Z-score according to the nomogram of pulmonary valve annulus [16].

### Statistical analysis

Categorical variables are presented as numbers with percentages, while continuous variables are presented as means with standard deviations or median with interquartile ranges (IQRs), according to the distribution of the data. The normality of data distribution was evaluated using the Shapiro−Wilk test. Kaplan−Meier survival estimates and the log-rank test were used for the analysis of time-dependent events and inter-group comparison. Cox proportional hazards modelling was fitted for the identification of univariable or multivariable risk factors for decreased time to adverse events. Variables with *P *<* *0.1 in the univariable analysis were used for the initial multivariable analysis, while variables with collinearity were analysed separately to ascertain the best-fitting statistical model. Stepwise backward elimination minimizing Akaike’s information criterion was used to determine significant risk factors in the multivariable analysis. A time-dependent receiver operating characteristics (T-ROC) curve was used to determine the optimal cut-off value [17]. Values of the area under the curve in the T-ROC curve at specific times (6-month intervals from 6 to 36 postoperative months) were used to compare the performance among the T-ROC curves. The optimal cut-off value was found at the particular postoperative timing after having a maximal Youden’s index in each T-ROC curve. Statistical outcomes with *P*-values ≤ 0.05 were considered significant. All analyses were performed using R software (version 3.6.3; R Foundation for Statistical Computing, Vienna, Austria).

## RESULTS

### Baseline characteristics

**Figure 1:**
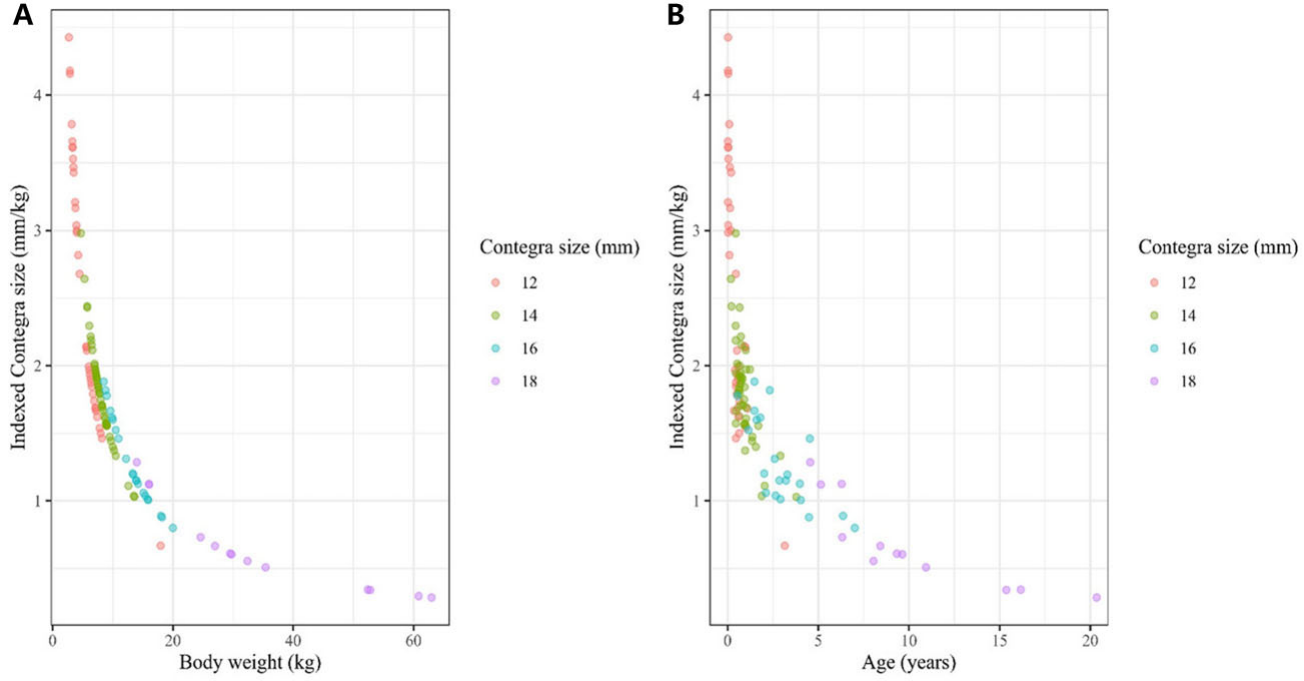
Correlation between (**A**) indexed Contegra size and body weight and (**B**) indexed Contegra size and age.

Of 115 patients in the study cohort, 54 were boys (54/115, 47%), and 11 were neonates at the time of implantation (11/115, 9.6%). The median Contegra diameter and indexed Contegra size were 14 mm (IQR 12–16) and 1.71 mm/kg (IQR 1.30–2.06), respectively. The entire baseline characteristics are summarized in Table 1. The indexed Contegra size significantly correlated with body weight (*P *<* *0.001; Fig. 1A) and age (*P *<* *0.001; Fig. 1B). The median postoperative stay in the intensive care unit was 4 days (IQR 2–12 days), and the median hospital stay was 12 days (IQR 8–20 days).

**Table 1:** Baseline characteristics in the entire study population

Variable	Result (*N* = 115)
Male sex	54 (47.0%)
Primary diagnosis	
TOF or its variants^a^	92 (80%)
With MAPCA	14 (12.2%)
With absent pulmonary valve syndrome	5 (4.3%)
Truncus arteriosus	11 (9.6%)
Aortic stenosis (with Ross procedure)	7 (6.1%)
Miscellaneous	5 (4.3%)
Underlying conditions	
Confluent pulmonary artery with normal arbourization	80 (69.6%)
History of previous cardiac operation	94 (81.7%)
Genetic anomaly	9 (7.8%)
McGoon ratio	2.1 (1.8–2.4)
Pulmonary artery index (mm²/m²)	259.9 (IQR: 193.8–332.6)
Characteristics at operation	
Age (months)	10.3 (IQR: 5.7–26.9)
Neonate	11 (9.6%)
Body weight (kg)	7.8 (IQR: 6.3–12.4)
BSA (m²)	0.39 (IQR: 0.33–0.55)
Conduit size (mm)	14.0 (IQR: 12.0–16.0)
12	36 (31.3%)
14	45 (39.1%)
16	21 (18.3%)
18	13 (11.3%)
Conduit diameter/body weight at operation (mm/kg)	1.71 (IQR: 1.30–2.06)
Conduit diameter (Z-score)^b^	1.6 (IQR: 0.9–2.3)
Dual RVOT pathway	11 (9.6%)
One-and-a-half ventricular repair	11 (9.6%)
Concomitant pulmonary artery angioplasty	67 (58.3%)
CPB time (min)	143 (IQR: 115–188)
ACC time (min) (in 106 patients)	52 (IQR: 36–93)

Numbers with percentages or medians with interquartile ranges are shown as appropriate.

a Tetralogy of Fallot, pulmonary atresia with ventricular septal defect, Fallot type double outlet right ventricle.

b Z-scores were calculated using the echocardiographic nomogram of the pulmonary valve annulus.

ACC: aortic cross-clamping.; BSA: body surface area; CPB: cardiopulmonary bypass; IQR: interquartile range; MAPCA: major aortopulmonary collateral arteries; RVOT: right ventricular outflow tract; TOF: tetralogy of Fallot.

### Survival, explantation-free survival and freedom from significant right ventricular outflow tract lesions

**Figure 2:**
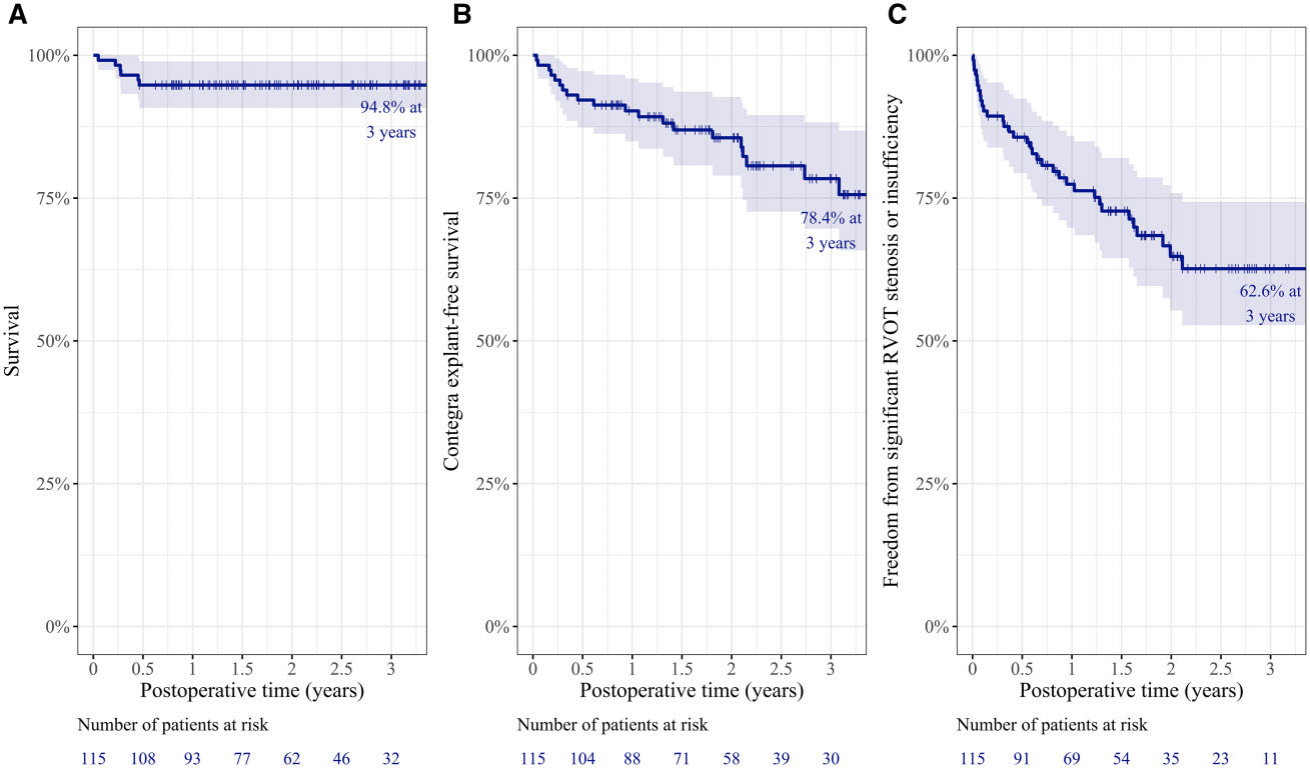
(**A**) Kaplan−Meier survival curve for the entire study population. (**B**) Contegra explantation-free survival curve. (**C**) Freedom from the development of right ventricular outflow tract lesions. Vertical ticks denote censoring and shaded areas represent a 95% confidence interval. RVOT: right ventricular outflow tract.

During the median follow-up duration of 25.1 months (IQR 14.8–37.7 months), there were 7 deaths (7/115, 6.1%), including 3 in-hospital deaths (2.6%). The overall survival rate at 3 years was 94.8% (Fig. 2A). Contegra explantations were performed for 15 patients. The indications for explantation were conduit endocarditis refractory to medical treatment in 4 patients, stenosis in 5 patients (conduit *per se* in 3 patients and distal to the conduit in 2 patients), Contegra valve regurgitation in 2 patients, valve regurgitation combined with distal stenosis in 2 patients and pre-emptive conduit replacement as part of operations for residual ventricular septal defect closure in 2 patients. The Contegra explantation-free survival rate at 3-year was 78.4% (Fig. 2B). Significant RVOT lesions developed in 34 patients. Among them, 20 patients had stenosis (conduit *per se* in 8 patients and at the distal anastomotic site in 12 patients), 8 patients had regurgitation, while 6 patients had significant valve regurgitation with distal anastomotic site stenosis. Freedom from the development of significant RVOT lesions was 62.6% at 3 years after initial implantation (Fig. 2C).

### Infective endocarditis in the Contegra conduit

Ten patients (10/115, 8.7%) developed infective endocarditis within the Contegra conduit: 8 patients with definite endocarditis and 2 patients with possible endocarditis, based on the modified Duke criteria [18]. The median duration from Contegra implantation to the development of infective endocarditis was 385 days (IQR 171–746 days) in the 8 patients diagnosed with definite endocarditis. All patients with definite endocarditis had vegetations on the Contegra valve, and additional vegetations were observed at the right pulmonary artery (*n* = 1), the left pulmonary artery (*n* = 1) and a patch used for ventricular septal defect closure (*n* = 1). Two patients with possible endocarditis and another 4 patients with definite endocarditis were successfully treated with antibiotics, while Contegra explantation was required for the remaining 4 patients due to severe stenosis in the conduit *per se*.

### Risk factor analysis for decreased time to adverse outcomes

In the univariable Cox analysis for decreased time to death or Contegra explantation, lower body weight, lower body surface, diagnosis of truncus arteriosus, history of previous cardiac operation, higher indexed Contegra size and higher Conduit diameter Z-score were identified. Still, only higher indexed Contegra size remained significant in the multivariable model (hazard ratio 2.32, 95% confidence interval 1.53–3.53, *P *<* *0.001, Table 2). In the univariable Cox analysis for decreased time to the development of significant RVOT lesions, male sex, younger age, lower body weight, lower BSA, diagnosis of truncus arteriosus, abnormal pulmonary arterial arbourization, history of previous cardiac operation, lower McGoon ratio, higher indexed Contegra size and higher Conduit diameter Z-score showed significant correlations. Multivariable Cox analysis, however, identified indexed Contegra size as the only risk factor for the development of significant RVOT lesions (hazard ratio 2.75 per 1 mm/kg increase, 95% confidence interval: 1.97–3.84, *P *<* *0.001, Table 3).

**Table 2:** Result of Cox proportional hazards ratio analysis for Contegra explantation-free survival

	Univariable			Multivariable		
	HR	95% CI for HR	*P*-value	HR	95% CI for HR	*P*-value
Male sex	1.42	0.60–3.39	0.43			
Age (months)	0.98	0.96–1.01	0.12			
Body weight (kg)	0.88	0.77–1.00	0.041	NA^c^		
BSA (m²)	0.11	0.009–1.51	0.099	NA ^c^		
Primary diagnosis						
TOF or its variants^a^	0.53	0.21–1.38	0.19			
With MAPCA	2.08	0.70–6.21	0.19			
With absent pulmonary valve syndrome	1.08	0.14–8.04	0.94			
Truncus arteriosus	2.59	0.87–7.73	0.088	NA^c^		
Aortic stenosis (with Ross procedure)	0.74	0.099–5.57	0.77			
Transposition of great arteries	2.23	0.30–16.82	0.44			
Other	NA		1.00			
Confluent pulmonary artery with normal arbourization	0.54	0.23–1.29	0.17			
History of previous cardiac operation	0.37	0.15–0.90	0.029	NA^c^		
Genetic anomaly	0.49	0.065–3.69	0.49			
McGoon ratio	0.59	0.28–1.27	0.18			
Pulmonary artery index (mm²/m²)	1.00	1.00–1.002	0.83			
Conduit diameter/body weight at operation (mm/kg)	2.32	1.53–3.53	<0.001	2.32	1.53–3.53	<0.001
Conduit diameter (Z-score)^b^	2.64	0.92–7.60	0.072	NA^d^		
Dual RVOT pathway	1.13	0.26–4.87	0.87			
One and a half ventricular repair	0.49	0.065–3.64	0.48			
PA arterioplasty upon Contegra implantation	0.60	0.25–1.41	0.24			
CPB time (min)	1.00	1.00–1.01	0.42			

a Tetralogy of Fallot, pulmonary atresia with ventricular septal defect, Fallot type double outlet right ventricle.

b Contegra diameter (Z-scores) were calculated using the echocardiographic nomogram of the pulmonary valve annulus.

c Not applicable in the final model because it had no significance in the multivariable model.

d Not applicable for multivariable analysis because Contegra diameter (Z-score) and indexed Contegra size had collinearity and the latter explains multivariable model better than the former.

BSA: body surface area; CPB: cardiopulmonary bypass; HR: hazard ratio; MAPCA: major aortopulmonary collateral arteries; NA: not applicable; PA: pulmonary artery; RVOT: right ventricular outflow tract; TOF: tetralogy of Fallot.

**Table 3:** Result of Cox proportional hazards ratio analysis for development of significant right ventricular outflow tract lesions

	Univariable			Multivariable		
	HR	95% CI for HR	*P*-value	HR	95% CI for HR	*P*-value
Male sex	1.84	0.92–3.67	0.085	NA^c^		
Age (months)	0.98	0.96–1.00	0.038	NA^c^		
Body weight (kg)	0.90	0.82–0.98	0.020	NA^c^		
BSA (m²)	0.030	0.002–0.39	0.008	NA^c^		
Primary diagnosis						
TOF or its variants^a^	0.72	0.33–1.60	0.42			
With MAPCA	0.78	0.24–2.55	0.68			
With absent pulmonary valve syndrome	1.22	0.29–5.08	0.79			
Truncus arteriosus	3.94	1.70–9.12	0.001	NA^c^		
Aortic stenosis (with Ross procedure)	NA		1.00			
Transposition of great arteries	1.17	0.16–8.65	0.88			
Other	NA		1.00			
Confluent pulmonary artery with normal arbourization	0.53	0.27–1.06	0.072	NA^c^		
History of previous cardiac operation	0.22	0.11–0.44	<0.001	NA^c^		
Genetic anomaly	0.71	0.17–2.95	0.63			
McGoon ratio	0.60	0.33–1.09	0.093	NA^c^		
Pulmonary artery index (mm²/m²)	1.00	0.99–1.00	0.52			
Conduit diameter/body weight at operation (mm/kg)	2.76	1.98–3.84	<0.001	2.75	1.97–3.84	<0.001
Conduit diameter (Z-score)^b^	1.82	1.02–3.27	0.043	NA^d^		
Dual RVOT pathway	1.21	0.43–3.43	0.72			
One-and-a-half ventricular repair	0.60	0.14–2.50	0.48			
PA arterioplasty upon Contegra implantation	0.88	0.45–1.73	0.71			
CPB time (min)	1.00	0.99–1.00	0.44			

a Tetralogy of Fallot, pulmonary atresia with ventricular septal defect, Fallot type double outlet right ventricle.

b Z-scores were calculated using the echocardiographic nomogram of the pulmonary valve annulus.

c Not applicable in the final model because it had no significance in the multivariable model.

d Not applicable for multivariable analysis because Contegra diameter (Z-score) and indexed Contegra size had collinearity and the latter explains multivariable model better than the former.

BSA: body surface area; CPB: cardiopulmonary bypass; HR: hazard ratio; MAPCA: major aortopulmonary collateral arteries; NA: not applicable; PA: pulmonary artery; RVOT: right ventricular outflow tract; TOF: tetralogy of Fallot.

### Optimal cut-off value of indexed Contegra size for right ventricular outflow tract lesions

**Figure 3:**
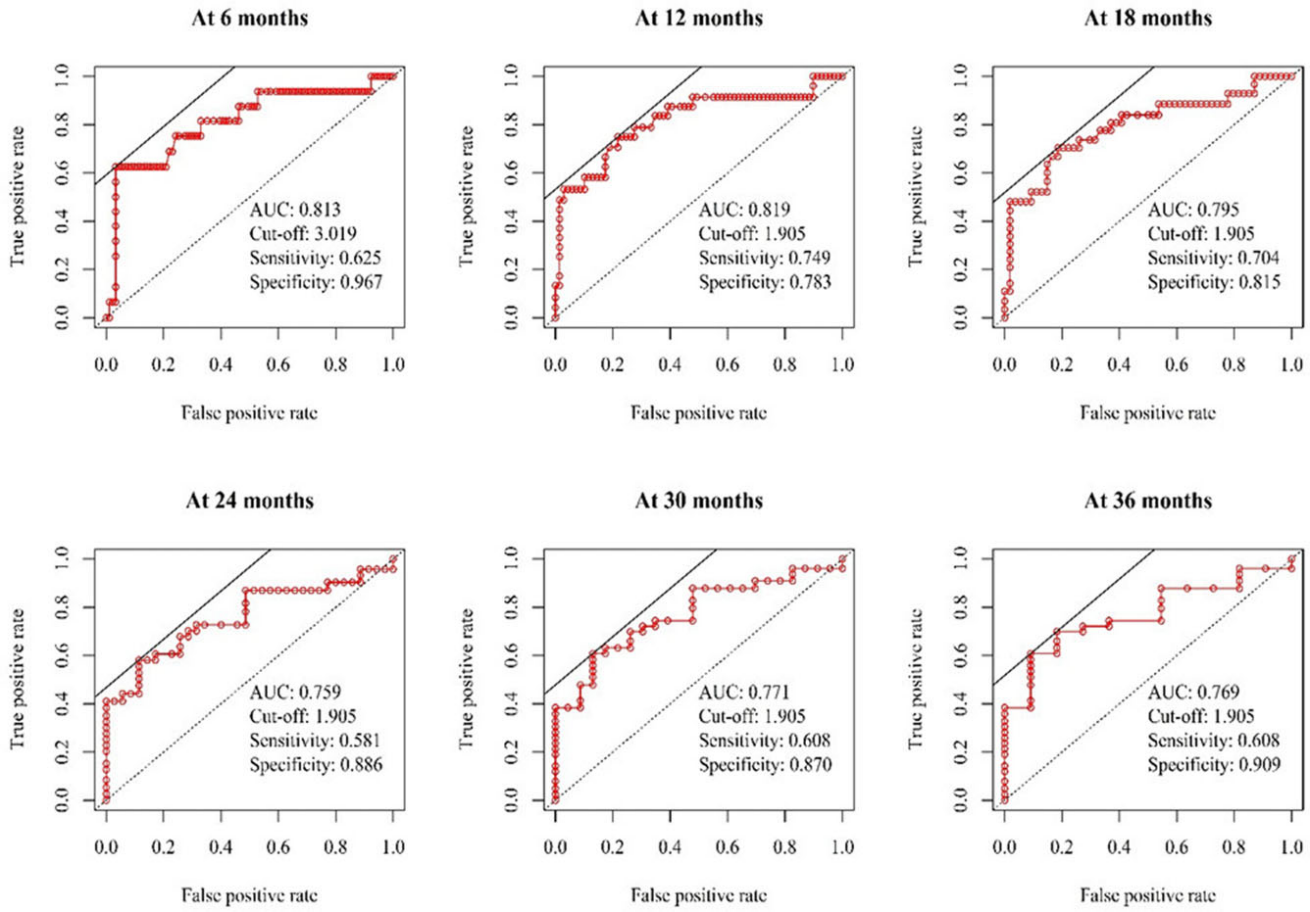
Time-dependent receiver operating characteristics curves for the development of significant right ventricular outflow tract lesions. The upper and lower dotted diagonal lines in each figures denote a random classifier and an identity line (or line of equality), respectively. AUC: area under the curve.

**Figure 4:**
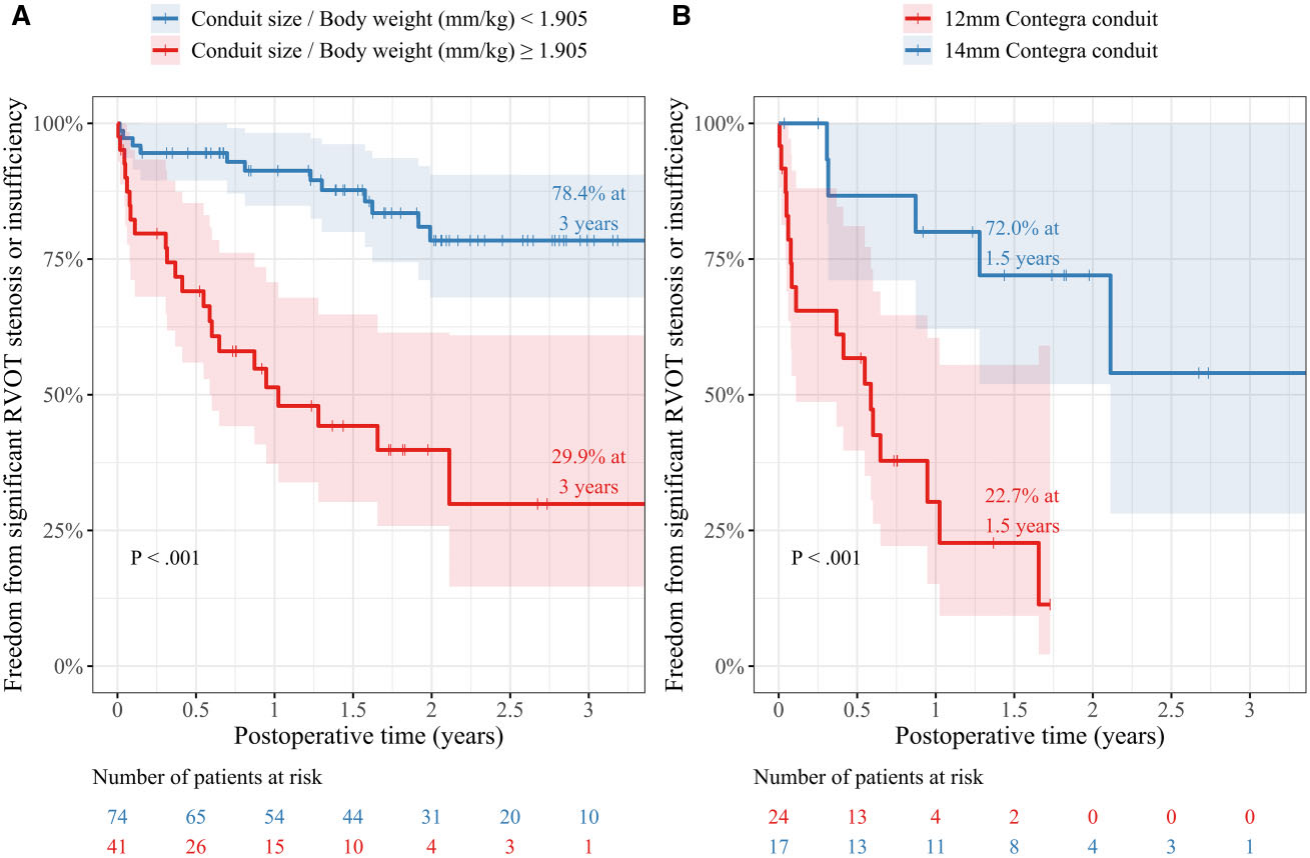
(**A**) Freedom from significant right ventricular outflow tract lesions in the entire cohort (*N* = 105) stratified by indexed Contegra size over and under 1.905. (**B**) Freedom from significant right ventricular outflow tract lesions in patients with oversized Contegra (indexed Contegra size ≥1.905, *n* = 41) stratified by graft diameter of 12 mm (unavoidable oversizing) versus 14 mm (intentional oversizing). Vertical ticks denote censoring and shaded areas represent a 95% confidence interval. RVOT: right ventricular outflow tract.

In the T-ROC curve analysis for indexed Contegra size predicting the development of significant RVOT lesions, we found the greatest area under the curve of 0.82 at 12 months after initial Contegra implantation. The indexed Contegra size cut-off value was 1.905 mm/kg at 12 months. The T-ROC curves reflecting the development of significant RVOT lesions according to the indexed Contegra size at post-implant 6, 12, 18, 24, 30 and 36 months are illustrated in Fig. 3. The entire study cohort was divided into 2 groups: 41 patients with indexed Contegra size ≥1.905 mm/kg and the remaining 74 patients with indexed Contegra size <1.905 mm/kg. The former group had a significantly lower rate of freedom from significant RVOT lesions than the latter group (29.9% vs 78.4% at 3 years, log-rank test: *P *<* *0.001; Fig. 4A). In the subgroup with oversized conduit (*n* = 41), Contegra diameter was 12 mm in 24 patients, who had unavoidably oversized conduits (because 12 mm conduits were the smallest conduits available), and 14 mm in 17 patients, who were deemed to have received intentionally oversized conduit (because 12 mm instead of 14 mm conduits could have been used). Patients with conduits that were unavoidably oversized had a significantly lower rate of freedom from significant RVOT lesions than patients with intentionally oversized conduits (28.0% vs 77.3% at 18 months, log-rank *P *<* *0.001; Fig. 4B).

## Discussion

This study demonstrated relatively promising outcomes of Contegra implantation in a consecutive series, even though the study cohort included 11 neonates (11/115, 9.6%) and 36 patients (36/115, 31.3%) who received the smallest Contegra conduits (12 mm in diameter). The clinical outcomes in the entire study population are comparable to the previous reports describing favourable outcomes [7, 8, 15, 19]. Several studies demonstrated comparable outcomes to other conduits, including homografts. Dave *et al.* [20] reported 10-year freedom from reintervention of 71% in 170 patients who had median age and body weight of 107 months and 23 kg. Also, a previous study by Falchetti and colleagues [21] showed no outcome difference between Contegra conduit and homograft in neonates. In contrast, other studies have reported adverse outcomes, including valve insufficiency [6], distal anastomotic site stenosis [9], thrombus and pseudoaneurysm formation [5, 10, 22] and endocarditis [4–6, 9, 11, 12, 19], have occurred in a considerable number of patients. In this study, Contegra valve regurgitation was observed in 14 patients, including 6 patients who developed concomitant supravalvar stenosis. Theoretically, an essential mechanism for Contegra valve insufficiency could be distension and aneurysmal changes from elastic degeneration and intimal hyperplasia [23] with valve coaptation failure, either by pulmonary arterial hypertension associated with pulmonary arterial arbourization anomaly or by distal anastomotic-side stenosis [8, 10, 22]. Similar to other Contegra studies [9], stenosis of the reconstructed RVOT turned out to be the predominant mechanism of significant RVOT lesions in this study. However, most of the stenotic lesions occurred in the distal anastomotic sites (*n* = 18), and stenosis of the conduit *per se* was relatively uncommon (*n* = 8), signifying that the development of stenotic RVOT lesions might have more to do with technical flaws upon distal anastomoses than with inherently defective graft material. Younger recipient age at implantation and use of smaller conduits have been reported as risk factors for the development of distal anastomotic site stenosis [4, 13–15]. Because younger patients tend to receive smaller conduits, suboptimal outcomes from younger patients could well result from smaller conduits. Thus, one would assume that the implantation of oversized conduits may lead to better conduit longevity. However, in a study investigating the impact of conduit size indexed to the body size on the durability and function of various conduits, both undersizing (Z-score of the conduit ≤ +1) and oversizing (Z-score of the conduit ≥ +3) were negatively associated with conduit longevity [14]. The exact mechanism of the deleterious effects of oversized conduits on graft function is uncertain. Size mismatch between the conduit and the confluent pulmonary artery may result in aneurysmal dilatation of the conduit, which may lead to further compression of the branch pulmonary arteries and acceleration of valve regurgitation [1, 14]. In the meantime, 8 patients had early RVOT failure, especially within 1 month in our study. All patients had 12 mm Contegra conduit, while 4 patients were neonates. Among them, 3 patients had isolated stenosis, 2 patients had pure regurgitation, and 3 patients had combined physiology. We thought that hypertensive pulmonary arterial pressure in small patients and a crowded mediastinal structure that complicates implantation technique could be a reason for poor early outcomes. Also, small dilatation of Contegra conduit by the pulmonary arterial pressure can cause more extensive RVOT obstruction than in other patient populations. An additional theoretical explanation could be that stenosis could be caused by increased wall shear stress and turbulent blood flow at the distal anastomosis site, caused by the kinking or abrupt decrease of pulmonary arterial diameter [24]. Therefore, oversized conduits should be avoided in young and small populations (i.e., body weight <6 kg). Additionally, we recommend minimizing intended oversizing, which would not provide a better outcome in relatively small patients (i.e. weight around 6 kg).

In our study, the main findings were that the oversizing of the Contegra conduit was associated with early RVOT failure in the entire study population. In the meantime, these outcomes could be affected by the patient’s body size and growth potential. We also had an investigation after excluding the patients who had 12 mm Contegra conduit implantation and the patients who had the inevitable use of 12 mm Contegra conduit; however, this association was not statistically significant in the subgroup analysis. Therefore, the result of our study should be used for the selection of high-risk patients among the entire population. The negative impact of intended oversized conduit in older patients should be reassessed in future studies.

The high incidence of associated infective endocarditis is another concern after Contegra implantation. Previous studies of conduit-related endocarditis have reported about a 10% cumulative incidence of infective endocarditis after implantation of bovine jugular vein conduits, including Contegra, which was significantly higher than homograft conduits [12, 19, 23, 25]. Although endocarditis can be managed by medical treatment, the probability of premature Contegra explantation after the development of endocarditis has been reported to range from 80% to 100% [11, 13]. In our study, 10 patients (10/115, 8.7%) developed definite (*n* = 8) or possible (*n* = 2) endocarditis based on the modified Duke criteria [18], and 4 of these patients underwent Contegra explantation. Careful surveillance for infection is required after Contegra implantation.

According to the observations of this study, alternative approaches may be required to minimize the use of oversized Contegra in high-risk populations. Given that the diameter of the smallest available Contegra is 12 mm, an alternative smaller conduit may be needed for neonates and young infants. A study regarding conduit durability after infantile implantation reported that the freedom from conduit replacement rates at 5 years after homograft, Contegra and Hancock valved conduit implantation were comparable [26]. Reported outcomes after implantation of any conduit for neonates are even worse [13], as observed in our study. Therefore, for high-risk patients, use of alternative conduits, such as expanded polytetrafluoroethylene hand-made valved conduit [27, 28], valveless polytetrafluoroethylene conduit, femoral homograft and pulmonary homograft, or employment of alternative surgical strategies, such as staged repair rather than early primary repair [29], should be considered to improve outcomes after RVOT reconstruction with conduit implantation.

### Limitations

Although this study statistically analysed the effects of each variable with multivariable analysis, the actual effects of age, Contegra diameter and diagnosis should be reviewed in future prospective studies. In particular, an investigation of RVOT reconstruction outcomes only in neonates or young infants should be performed to determine the effects of each risk factor. Comparative studies between Contegra and hand-made expanded polytetrafluoroethylene conduits are also warranted to clarify the optimal conduit choice for high-risk patients. Also, the retrospective single-centre study nature makes the results less generally adopted. And follow-up duration was short since the implantation of the Contegra conduit was started in 2016 because of local availability. Therefore, further follow-up is required given the small study population and events during the limited follow-up period.

## CONCLUSION

After Contegra implantation for RVOT reconstruction, the outcomes were acceptable, allowing its application in various clinical settings. Early failure of the conduit *per se* was uncommon. However, infective endocarditis in implanted conduits was not uncommon, and the use of oversized conduits, which is unavoidable in small patients, is a risk factor for early failure.

**Conflict of interest:** none declared.

## Data Availability

The article’s data will be shared on reasonable request to the corresponding author.

## ABBREVIATIONS

IQR: Interquartile range
RVOT: Right ventricular outflow tract
T-ROC: Time-dependent receiver operating characteristic
